# Supplementary material for: Central dogma rates and the trade-off between precision and economy in gene expression
Source: Nat Commun. 2019 Jan 8;10:68. doi: 10.1038/s41467-018-07391-8 (PMC6325141; doi:10.1038/s41467-018-07391-8)
Supplement: Supplementary file 1 — Supplementary Information [file 41467_2018_7391_MOESM1_ESM.pdf]

Supplementary Information

Central dogma rates and the trade-off between precision and economy in gene expression

Hausser et al.

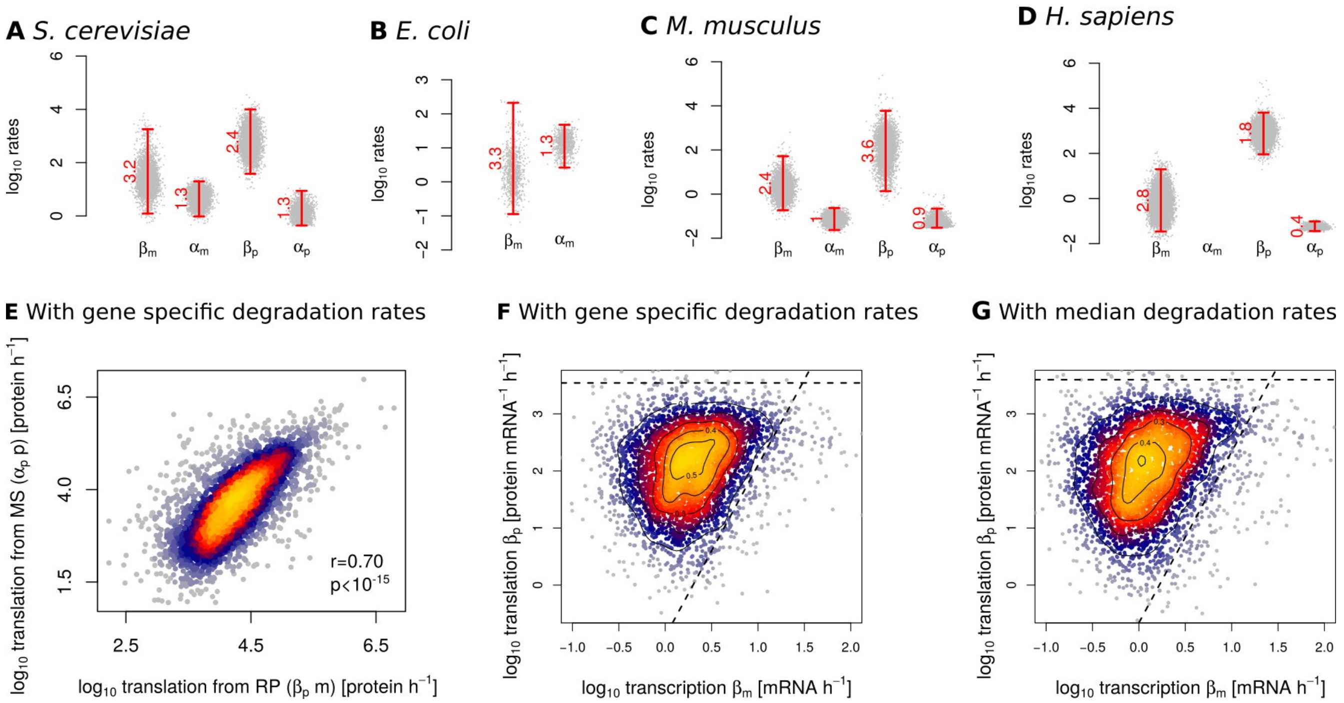

Supplementary Figure 1. **A – D.** Distribution of transcription, translation and mRNA and protein decay rates in four model organisms. **A.** *S. cerevisiae*: transcription and translation rates from Weinberg et al.[1], mRNA decay rates from Eser et al.[2], protein decay rates from Belle et al.[3]. **B.** *E. coli*: transcription rates from Li et al.[4], mRNA decay rates from Chen et al.[5]. **C.** *M. musculus*: all rates from Schwanhüusser et al.[6]. **D.** *H. sapiens*: transcription and translation rates from Eichhorn et al.[7], protein decay rates from Cambridge et al.[8]. Rates were estimated as described in the Methods. Bars span the range from the 0.5% quantile to the 99.5% quantile of rates (i.e. 99% of genes). **E – G.** Taking into account the specific mRNA and protein decay rates of each gene has a negligible effect on the distribution of genes in the Crick space. **E.** Translation rates estimated from ribosome profiling densities correlate well with translation rates estimated from measurements of protein abundance and decay. P-value from Pearson's test. Data: ribosome profiling in *M. musculus* 3T3 cells from Weinberg et al.[1], protein abundance and decay rates from Schwanhüusser et al.[6]. **F.** Transcription and translation rates as estimated from measurements of mRNA and protein abundances and decay by Schwanhüusser et al.[6]. **G.** Same as E., except that transcription and translation rates where estimated from mRNA and protein abundance by setting mRNA and protein decay rates to their median. Gene positions in panels E and F differ by 0.3 (root mean square deviation in $\log_{10}$ rates), which is small (10%) compared to the dynamic range of transcription and translation rates which vary over three orders of magnitude.

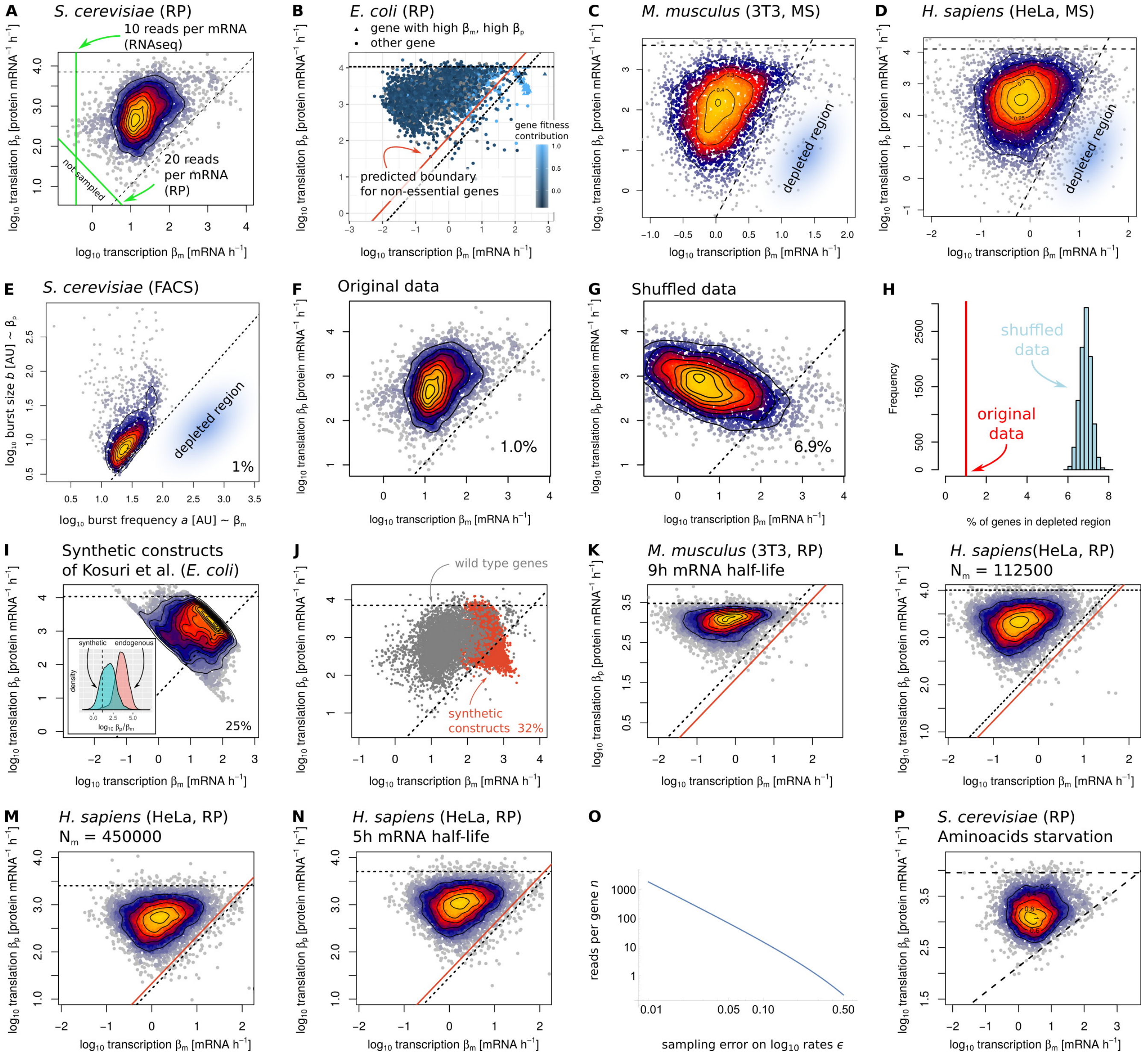

Supplementary Figure 2. A. The ’not sampled’ region is explained by sequencing depth and our focus on genes with at least 10 mRNAseq reads and 20 ribosome profiling (RP) reads per mRNA (two green lines). **B.** In *E. coli*, a group of 62 genes (triangles) contribute strongly to fitness. Excluding these genes, the boundary of the depleted region (diagonal dotted black line) has intercept $\log_{10} k = 1.7 \pm 0.1$, which is higher than $\log_{10} k = 1.1 \pm 0.1$ found using all genes. A higher intercept is expected for non-essential genes (Methods). This is illustrated by the red line, which represents the boundary predicted for genes that contribute 1% of the organism’s fitness. Fitness data: Baba et al.[9]. (continued on the next page)

Supplementary Figure 2. **C – E.** The depleted region is found in the mass spectrometry (MS) and mRNAseq data of Schwanhüusser et al.[6] (panel C) and Nagaraj et al.[10] (panel D), as well in the flow cytometry data of Newman et al.[11] (panel E). By estimating transcription and translation rates from the mean protein abundance and coefficient of variation[12,13], we find a depleted region whose boundary has slope 1 (panel E), as in ribosome profiling datasets. The boundary of the depleted region in the two proteomics datasets has slope larger than one ($\beta_p \sim \beta_m^4$). The different slope observed with mass-spectrometry compared to ribosome profiling and flow cytometry datasets could be explained by technical limitations in these pioneering mass-spectrometry datasets and differences in the error structure of mRNAseq and mass-spectrometry datasets[14]. **F – H.** Genes with high β_m and low β_p are statistically depleted. **F.** The boundary of the depleted region is a line of slope 1 such that 99% of genes are above the line. *S. cerevisiae* data from Weinberg et al.[1]. **G.** Shuffling β_m and β_p while keeping the marginal distributions of β_p and protein abundance constant increases the fraction of genes located in the depleted region. **H.** All 10^4 re-shuffled sets of β_m and β_p show more genes in the depleted region. Thus, the depletion of this region is statistically significant ($p < 10^{-4}$). Repeating this procedure in *E. coli*, *H. sapiens* and *M. musculus* leads to the same conclusion ($p < 10^{-4}$). **I.** 25% of the synthetic gene constructs of Kosuri et al.[15] fall in the depleted region of Crick space. The median β_p/β_m ratio is much lower for synthetic genes compared to endogenous genes (10^2 vs $10^{3.5}$, $p < 10^{-15}$ at Mann-Whitney test). 25% of synthetic genes are in the depleted region vs 1% of endogenous genes. **J.** In *S. cerevisiae*, 32% of 4172 synthetic constructs of Sharon et al.[16] fall in the depleted region of Crick space. Transcription and translation were estimated from abundance and noise measurements[17] collected by flow cytometry. **K – N.** The depleted region and the predicted boundary of this region are robust to uncertainties in mRNA half lives and in total number of mRNAs per cell N_m. **O.** The error on $\log_{10}$ mRNA abundance ε can be controlled by discarding genes with low number of reads n. **P.** In conditions of slower growth such as amino-acids starvation in *S. cerevisiae*, we also find a depleted region in Crick space. The boundary has slope 0.5 instead of 1, indicating perhaps a different tradeoff. Ribosome profiling data from Ingolia et al.[18].

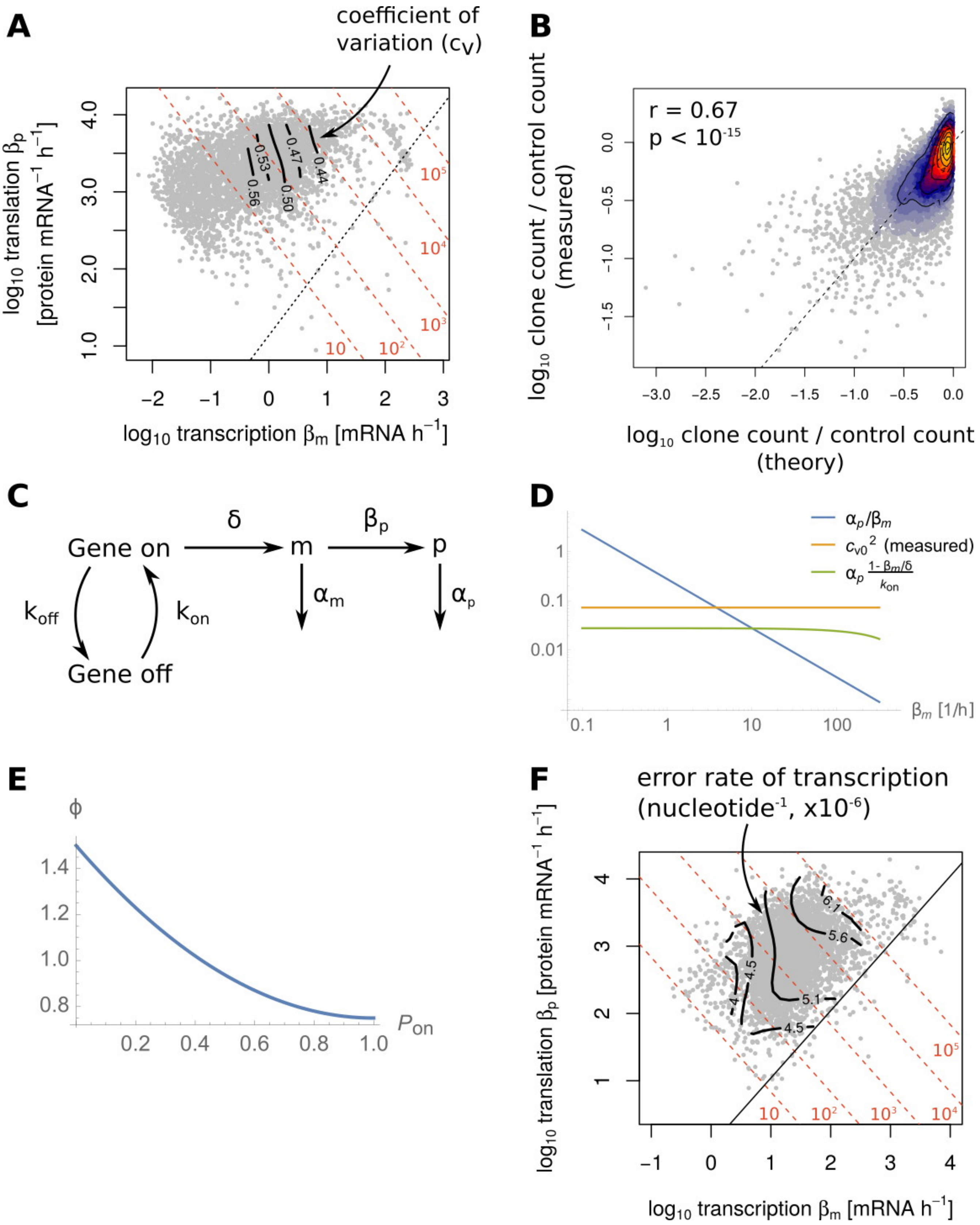

Supplementary Figure 3. See legend on the next page.

Supplementary Figure 3. **A.** In *E. coli*, coefficients of variation scale with transcription rates. Transcription and translation rates inferred from Li et al.[4]. CVs from Taniguchi et al.[19]. **B.** Clone abundance in growth competition experiments can be predicted from theoretical estimates of fitness cost of mRNA c_m and fitness cost of protein c_p. We assumed a growth time $t = 24$h in this figure. The correlation between predictions and measurements is independent of the growth time t (Methods). The dotted line represents a perfect fit between measurements and experiments ($y = x$). **C.** The 3-stage telegraph process can be used to models how intrinsic noise in gene expression is affected by different reaction rates. **D.** In the 3-stage model of gene expression of panel C, intrinsic protein noise is mainly due to small mRNA copy number noise (blue line) when β_m is small. For large β_m, intrinsic protein noise is dominated by gene activation noise (green line), which is almost constant for β_m in physiological range (x-axis). Constants are from the experiments of So et al.[20] and Taniguchi et al.[19]: $\alpha_p = 0.28\text{h}^{-1}$, $c_{v0}^2 = 0.07$, $\delta = 800\text{h}^{-1}$, $k_{on} = 10\text{h}^{-1}$. With these constants, the noise floor observed experimentally (orange line) is larger than the gene activation noise. **E.** By neglecting gene activation dynamics, one can estimate the coefficient of variation from the central dogma rates with an error ϕ less than 1.5-fold. Note however that this result does not hold for highly expressed genes (see Methods). **F.** The error rate of transcription is approximately constant across Crick space. Error rates from Gout et al.[21], transcription and translation rates from Weinberg et al.[1].

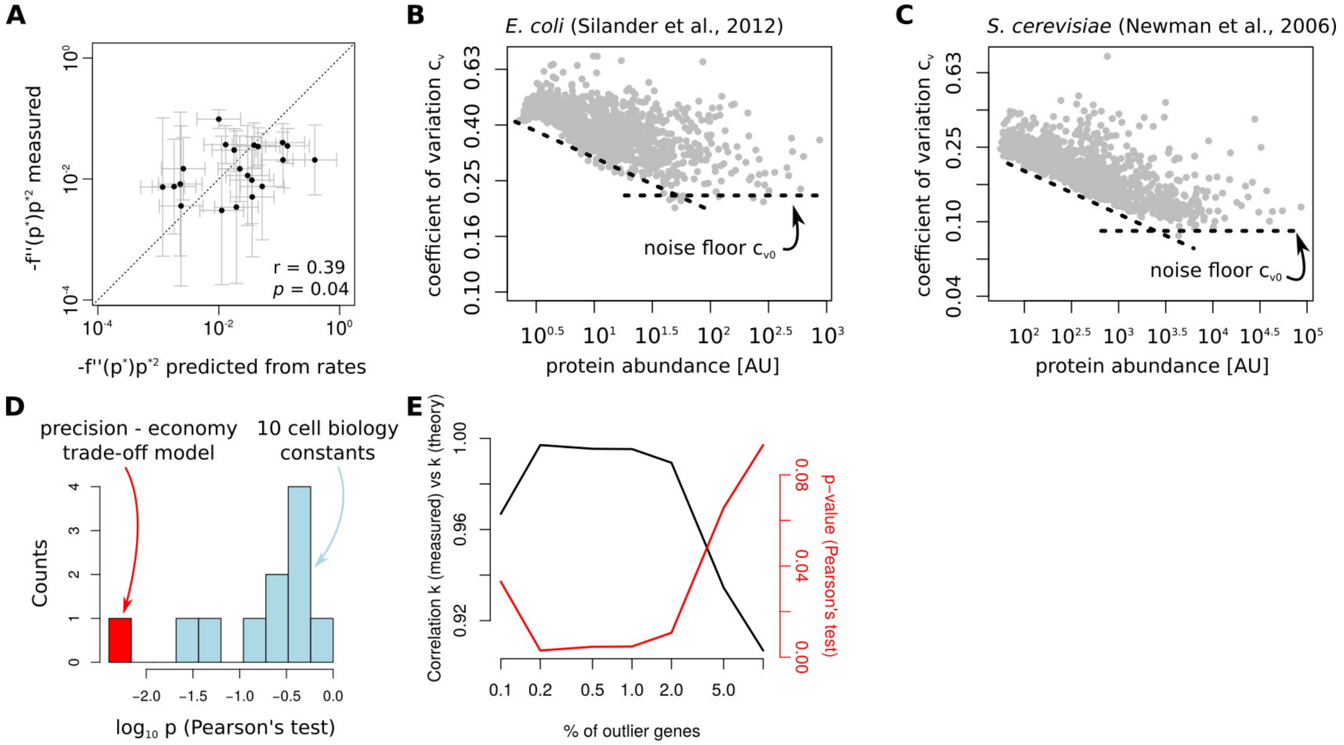

Supplementary Figure 4. **A.** Measured curvatures of fitness functions are within measurement error of curvatures predicted by theory from transcription and translation rates. Curvatures were estimated from the experiments of Keren et al.[22] who measured fitness as a function of log protein abundance. The quantity $f''(p^*)p^{*2}$ can be directly estimated from this data (Supplementary Methods). We used the precision - economy theory to predict $f''(p^*)p^{*2}$ from the central dogma rates (Methods). Error bars represent standard errors. **B.** A noise floor $c_{v0} = 0.22 \pm 0.01$ is found in the measurements of the protein noise conferred by *E. coli* promoters of Silander et al.[23]. This is comparable to the noise floor $c_{v0} = 0.27 \pm 0.01$ found in the measurements of Taniguchi et al.[19] (Fig. 4C). **C.** A noise floor c_{v0} is also found in *S. cerevisiae*. Coefficients of variation and protein abundance from Newman et al.[11].
D. Individual constants cannot predict the position k of the boundary of the depleted region. We correlated the position of the boundary ($\log k$) of the depleted region with 1. the predictions from the theory (Eq. 5 of the main text) and 2. the 8 cell biology constants listed in Table 1 (number of mRNAs N_m and protein N_p per cell, cell volume V, growth rate μ, active protein decay rate α_{deg}, effective protein decay α_p, mRNA decay α_m, noise floor c_{v0}), plus the maximal translation rate β_p^{max} and total transcription output $\sum \beta_m$ (Table 1). We tested for significant correlations between these constants (in log) and measured $\log k$ using Pearson's test. Measured $\log k$ correlates significantly with the predictions of the theory. No significant correlation is found between any of the 10 cell biology constants and measured $\log k$. **E.** The correlation between measurements and theory is robust to varying the fraction of genes used in defining the depleted region from 0.2% to 2% (10-fold). P-values were computed by comparing $\log k$ from theory and $\log k$ from measurements using Pearson's test.

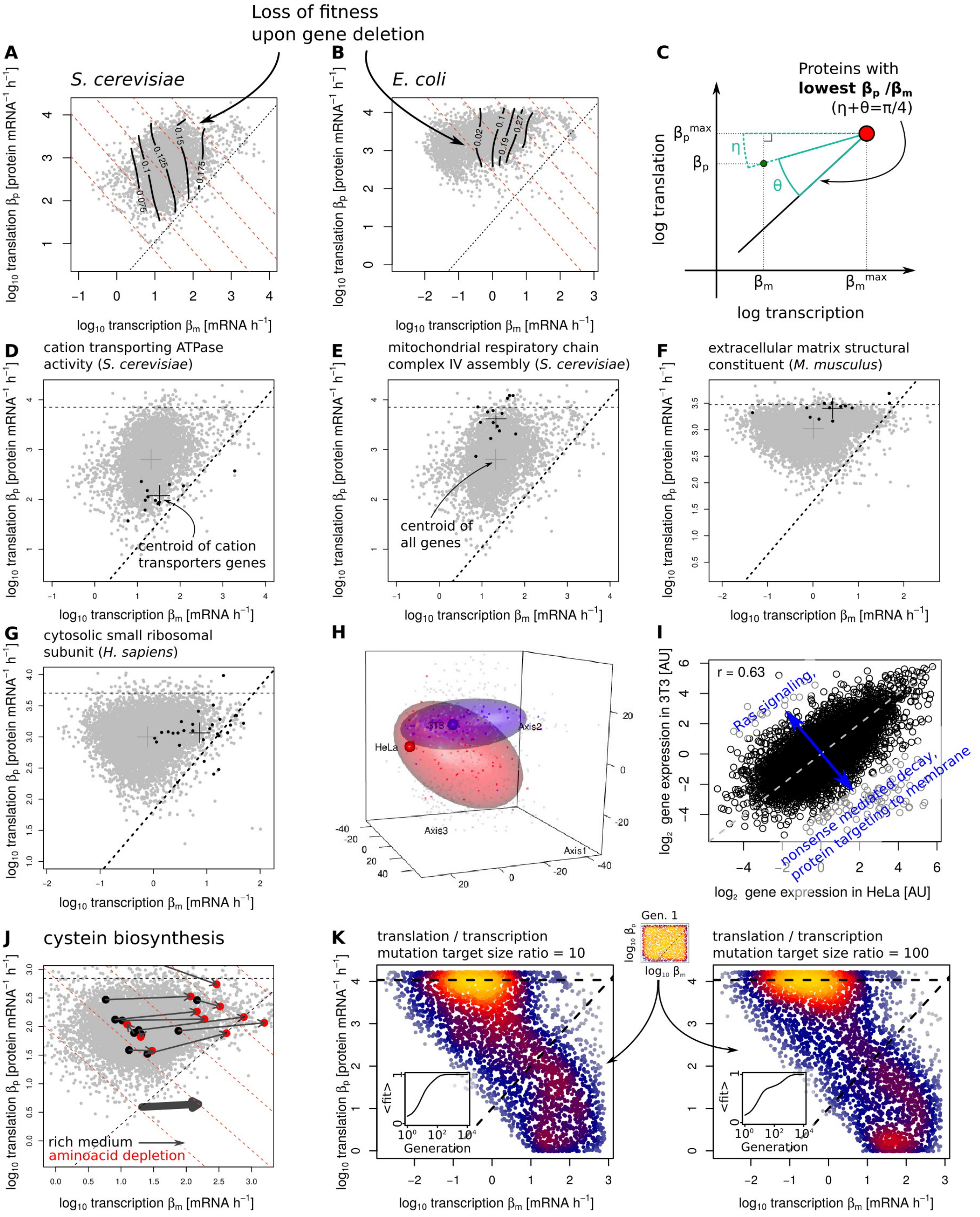

Supplementary Figure 5. See legend next page.

Supplementary Figure 5. **A – B.** Genes located close to the boundary of depleted region provide more fitness benefit than genes located far from it. Contours of fitness loss upon gene deletion in *S. cerevisiae* (panel A) and *E. coli* (panel B). We obtained fitness data from Steinmetz et al.[24] (*S. cerevisiae*) and Baba et al.[9] (*E. coli*). Fitness was Gaussian-smoothed using the same procedure as the CV data (Methods). **C.** To test for statistical associations between the function of genes and their position in the Crick space, we describe each gene by an angle θ. $\theta = 0$ for genes with lowest possible translation / transcription, whereas genes that maximize the translation / transcription ratio have $\theta = \pi/4$. θ can be computed from a gene's β_m and β_p, and from the maximal transcription and translation rates β_m^{max} and β_p^{max}. **D – G.** Genes with different functions (GO categories) are found close or far from the boundary of the depleted region. In each panel, black dots represent genes belonging to a specific group of genes. **H.** Principal component analysis of gene expression in 859 cancer cell lines (gray dots) shows that HeLa cells cluster with ovarian cell lines (red dots) whereas 3T3 cells cluster with skin cell lines (blue dots). Ellipses represent the covariance of skin and ovarian cell lines at the 75% confidence level. **I.** 3T3 cells over-express Ras signaling genes whereas HeLa cells over-express genes involved in nonsense mediated decay and protein targeting to membrane (Gene Set Enrichment Analysis, see Supplementary Methods). **J.** Upon a sudden depletion of amino-acids, abundance of cystein biosynthesis proteins increases by up-regulation of transcription, which re-positions genes in the region of Crick space associated to high precision. Ribosome profiling data from Ingolia et al.[18]. **K.** Evolutionary simulations suggest that difference in the target size of mutations that affect transcription and translation cannot explain the depleted region of Crick space. See methods section 'Simulating different target size of mutations affecting transcription and translation'.

Organism	N_m	V	N_p	μ	α_{deg}	α_p	α_m	c_{v0}
S. cerevisiae	60000	37	1.1×10^8	0.42	0.92	1.3	5.1	0.1
M. musculus (3T3)	180000	2000	6.0×10^9	0.03	0.01	0.04	0.14	0.3
H. sapiens (HeLa)	230000	2500	7.5×10^9	0.03	0.02	0.05	0.06	0.3
E. coli	1400	1	3×10^6	1.9	0	1.9	15	0.25

Supplementary Table 1. Constants used to compute transcription and translation rates from mRNAseq, ribosome profiling and proteomics data. Listed are values for the number of mRNAs per cell N_m, cell volume V [μm^3], number of proteins per cell N_p, growth rate μ [h^{-1}], protein degradation rate α_{deg} [h^{-1}], protein decay rate $\alpha_p = \alpha_{deg} + \mu$ [h^{-1}] , mRNA decay rate α_m [h^{-1}] and noise floor c_{v0}. References for data sources are detailed in the Methods.

Organism	low β_p/β_m	high β_p/β_m
E. coli	ribosome, ATP synthesis	response to oxidative stress, salvage of metabolites
S. cerevisiae	glycolysis, pumping out protons, chromosome separation, tRNA aminoacylation, growth signaling (Ras, TOR), 90S pre-ribosome	respiration
M. musculus (3T3)	translation initiation, cytoskeleton, Ras signaling	cell differentiation, collagen, extracellular matrix, chromatin
H. sapiens (HeLa)	ribosomal subunits, translation, nonsense mediated mRNA decay	

Supplementary Table 2. Genes that are key to growth tend have low β_p/β_m whereas genes needed for stress response, survival and differentiation have high β_p/β_m. For each of the four organisms studied in the present study, the table lists GO categories that are significantly enriched (FDR < 0.01) at high and low β_p/β_m.

Genes potentially involved in bet-hedging strategies have high β_p/β_m. For example, in *E. coli*, oxidative stress response induces tolerance to antibiotics and persistence[25,26]. Persistence is a bet-hedging strategy used by bacteria to cope with unpredictable environments[27]. Consistent with their role in regulating persistence, oxidative stress response genes have high β_p/β_m. In *S. cerevisiae*, a single-cell screen previously identified respiration genes as potential bet-hedging genes[28]. Respiration provides tolerance to glucose starvation at the expense of a slower growth rate[28–31]. Consistent with their possible role in hedging glucose starvation, respiration genes have high β_p/β_m (Supplementary Fig. 5E).

Differences in GO terms with high and low β_p/β_m in human and mouse can be explained by differences in the underlying biology of the cell lines. For example, genes involved in translation initiation, ATP binding, RNA binding as well as ubiquitin-dependent protein catabolism appear both in 3T3 and HeLa cells. Other groups of genes are only found in one of the two cell lines. Such difference in GO terms is expected for cancer cell lines of different tissue origin: 3T3 cells cluster with skin cell lines whereas HeLa cells cluster with ovary cell lines (Supplementary Fig. 5H, Supplementary Methods). Differences in genes expressed by 3T3 and HeLa cells can explain differences in genes with high and low β_p/β_m (Supplementary Methods). For example, 3T3 over-express Ras signaling genes (Supplementary Fig. 5I). These genes have significantly low β_p/β_m in 3T3 cells but not in HeLa (Supplementary Data 1). In contrast, HeLa cells over-express genes involved in nonsense mediated decay and SRP-mediated protein targeting to membrane (Supplementary Fig. 5I) which have low β_p/β_m in HeLa but not in 3T3 (Supplementary Data 1).

Supplementary Discussion

This study is based on an optimality approach to evolutionary biology[32] whose aim is to explain adaptations found in living organisms in terms of selective forces. This approach has been used to explain, for example, bacterial growth laws[33,34] or energy landscape in molecular recognition[35]. It is a fruitful approach in the

sense that it can suggest new experiments (see discussion).

The quantitative model rests on the assumption that transcription and translation rates can be tuned independently. Although coupling is seen in bacteria[36] and eukaryotes[37], that coupling has itself evolved rather than being an absolute constraint. Studies on synthetic promoters and ribosomal binding sites in *E. coli* indicate that transcription and translation rates can be changed independently over a wide range[15]. In *S. cerevisiae* too, flow-cytometry analysis of synthetic gene constructs suggest that the eukaryotic gene expression machinery can achieve transcription and translation rates that position genes in the depleted region of Crick space (Supplementary Fig. 2J).

Another coupling relevant to the present study is co-translational stabilization of mRNA[38]. Since rapid translation stabilizes mRNA and mRNA abundance is used to estimate transcription, our estimates of transcription and translation are statistically coupled. However, variation in co-translation stability is smaller than gene-gene variation in transcription rates by about three orders of magnitude: co-translational stability varies by less than one order of magnitude[38], compared to 3 orders of magnitude for transcription. Therefore, our estimates of transcription and translation are not formally independent, but the dependence is small.

Our theory assumes that cells are well-adapted to the conditions in which transcription and translation were measured, namely rapid growth in rich medium. Rapid growth is thought to be a key fitness component of microorganisms like *E. coli* and *S. cerevisiae*[39,40]. Rapid growth also occurs in several contexts in mammals, including immune expansion, cancer, development and stem cells in tissues with rapid turn-over. The mammalian HeLa and 3T3 cell lines studied here have likely been selected for fast growth. HeLa cells were collected from cervical cancer, a condition which selects for mutations that provide a growth advantage. Following collection, HeLa cells underwent serial dilutions for 4 months[41], a procedure which selects for growth[40]. In the case of 3T3 cells, embryonic fibroblasts were collected and underwent serial dilutions for 3-4 weeks[42]. In the process, cell growth first collapses due to cell senescence, and then recovers to levels of freshly collected embryonic fibroblasts, presumably because immortalized mutants take over the population. Thus, both HeLa and 3T3 cells are likely well adapted to the conditions in which transcription and translation were measured, namely rapid growth in rich culture medium. Note that these growth conditions may not represent the healthy environment of mammalian

cells in vivo, but rather the growth conditions found in a tumor or in cell culture.

Here we focused on growing cells because there is ample data covering mRNA and protein abundance and decay as well as translation dynamics through ribosome profiling. In conditions of slower growth such as amino-acids starvation in *S. cerevisiae*[18], we also find a depleted region in Crick space (Supplementary Fig. 2P). The boundary has slope 0.5 instead of 1, indicating perhaps a different tradeoff. Identifying this tradeoff could be an excellent follow-up study.

We proposed here that a tradeoff between economy and precision in protein abundance explains the depletion of genes with high transcription and low translation. However, the rate of transcription and translation could also impact protein sequence accuracy. For example, the risk that a transcriptional error propagates to many proteins could be reduced by increasing or decreasing transcription, leading to potential tradeoffs between sequence accuracy, precision and economy. At present, tradeoffs involving protein sequencing accuracy are not supported by the data. Measurements of error rates for thousands of genes in *S. cerevisiae* show no correlation between transcription error rates and transcription rates[21]. Variations in error rates over Crick space are found in a narrow range between 4.0×10^{-6} nucleotide^{-1} and 6.1×10^{-6} nucleotide^{-1}, with no clear correlation with transcription, translation or the translation / transcription ratio (Supplementary Fig. 3F). Thus, considerations of protein sequence accuracy are unlikely to deplete genes with high transcription and low translation rate.

Another possible hypothesis to explain the depleted region of Crick space is that mutations that tune protein abundance could affect transcription and translation with different frequency. Difference in the frequency of mutations affecting transcription and translation could stem from a difference in mutational target size. To explore this hypothesis, we simulated evolution towards a given protein abundance (drawn form the natural protein abundance distribution) by mutations that affect transcription and translation rates with different mutational targets (see Supplementary Methods). We find that different mutation target size ratios do not create a depleted region (Supplementary Fig. 5K). The distribution in simulated Crick space is along a diagonal that is nearly orthogonal to the distribution of natural gene. We conclude that target size alone is not a viable explanation for the depleted region.

Our theory focuses how on considerations of precision and economy set transcription and translation rates. Yet, precision in gene expression depends not only on the transcription rate but also on the epigenetic

environment which affects the transcription mode — burst size, burst frequency[43,44]. As a result, two genes with the same transcription rate may exhibit different noise.

The transcription mode does not affect our predictions of the boundary of the depleted region of Crick space because the formula for the boundary of the depleted region (Eq. 5 of the main text) does not depend on the transcriptional mode of the gene, only on the noise floor c_{v0}.

The transcription mode has a only small effect on the optimal translation / transcription ratio. The reason is that most of the noise in gene expression can be described by combining transcription, protein abundance and extrinsic noise (Eq. 3 of the main text). While the epigenetic environment of individual genes can cause deviations from the formula for c_v^2 of Eq. 3 (main text), we estimate that these deviations are smaller than 50% (see section 'Protein fluctuations as a function of the central dogma rates' in the Methods). This estimate is consistent with the observation that knocking out epigenetic regulators impacts burst size and frequency by less than 30% on average[44]. A 50% error on the c_v^2 translates to a 50% error on the optimal translation/transcription ratio (see Eq. 54 and Eq. 55 in the main text). This error is small compared to the 3 orders of magnitude variation in transcription and translation rates.

Supplementary Methods

Power-law scaling of mRNA Fano factor with mRNA abundance cannot explain the noise floor

In *E. coli*, So et al.[20] observed that the Fano factor of mRNA scales as a power-law of mRNA abundance m,

$$\frac{\sigma_m^2}{m} = 1 + s m^{\gamma} \tag{1}$$

with $\gamma \simeq 0.64$ and $s \simeq 1.5$. This scaling holds for mRNAs whose abundance range from 0.3 to 40 to per cell[20]. A similar scaling was observed in a human fibroblast cell line[5].

Here we ask whether this scaling can predict the noise floor, which would remove the need for the phenomenological factor c_{v0}^2 in the expression for the protein noise c_v (Eq. 3 of the main text).

In the case of the stochastic model of gene expression of the previous section (Supplementary Fig. 3C), Paulsson[45] showed that the Fano factor for mRNA abundance m can we written as

$$\frac{\sigma_m^2}{m} = 1 + \alpha_m m \frac{k_{off}}{k_{on}(\alpha_m + k_{off} + k_{on})}. \tag{2}$$

By equating the observed Fano factor power-law (Eq. 1) and the theoretical expression for the Fano factor (Eq. 2), we find how the gene activation noise term varies with the transcription rate β_m:

$$\alpha_m \frac{k_{off}}{k_{on}(k_{off} + k_{on} + \alpha_m)} = s \left(\frac{\beta_m}{\alpha_m}\right)^{\gamma - 1} \tag{3}$$

where we have used $m = \beta_m / \alpha_m$. Plugging this expression into the expression for c_v^2 derived by Paulsson[45] (Eq. 32 of the main text), we can find how protein noise c_v^2 varies with transcription rate β_m

$$c_v^2 = \frac{1}{p} + \frac{\alpha_m \alpha_p}{\alpha_m + \alpha_p} \left[\frac{1}{\beta_m} + \frac{s}{\beta_m} \left(\frac{\beta_m}{\alpha_m}\right)^{\gamma} \left(1 + \frac{\alpha_m}{k_{off} + k_{on}}\right) \right]. \tag{4}$$

For $\gamma < 1$ and in the typical case in which gene (in-)activation is fast compared to mRNA decay $k_{off}, k_{on} \gg$

α_m, c_v^2 goes to 0 for large p and β_m. Thus, there is no noise floor in this regime.

In the opposite regime where mRNA decay is fast compared to gene (in-)activation $\alpha_m \gg k_{off} + k_{on}$, we could have $k_{on} \sim \beta_m$ or $k_{off} \sim \beta_m^{-1}$. In the first case, c_v^2 goes to 0 for large p and β_m, so there is no noise floor. We already studied the second case in the previous section on *E. coli* noise and found that the resulting gene activation noise is too small compared to the observed noise floor. Finally, plotting c_v^2 as a function of β_m for typical values of α_m, α_p, s, k_{on}, k_{off} and γ, we confirmed that the Fano factor power law could not explain the noise floor observed in single cell experiments.

In conclusion, incorporating the Fano factor power law (Eq. 1) into the model, we find that it results in no noise floor for $\gamma \simeq 0.5$ or in a noise floor that is too low compared to experimental observations. Since the noise floor is a well-founded experimental observation[11,19,23,46], the power law by itself cannot explain the full noise behavior. Additional biology, such as extrinsic noise or increased transcriptional bursting at large transcription rates, is needed to understand the noise floor.

Estimating the curvature of fitness functions from the measurements of Keren et al.

Keren et al.[22] measured the fitness of *S. cerevisiae* cells as a function of log-expression for growth in glucose. Specifically, these measurements map $\log_{10}$ gene expression x to fitness $f(x)$, with $x = \log_{10} p$. These measurements do not allow to estimate the curvature of the fitness function $f''(p^*)$ directly. Nevertheless, they allow to estimate a closely related quantity, namely:

$$\frac{d^2 f}{dx^2} = \frac{d^2 f}{d(\log_{10} p)^2} \simeq \log(10)^2 p^2 f''(p), \tag{5}$$

where we neglected $f'(p)$ since we expand around the fitness optimum. In this section, we compare the measured $p^2 f''(p)$ to the predictions from the theory.

We focused on genes present in both the study of Keren et al.[22] and the ribosomal profiling data of Weinberg et al.[1]. To have enough context to estimate the local curvature, we considered only genes that could be both under-expressed and over-expressed compared to their wild-type expression by at least half an order of magnitude. Following Keren et al.[22], we also discarded low-quality genes, such as genes whose fitness value at wild-type expression was significantly lower than the fitness of the wild-type. This left 34 genes for analysis. We further excluded 9 genes with TATA promoters because these genes

tend to have large transcriptional burst size[47]: our model assumes a small transcriptional burst size and hence cannot accurately model the noise of genes with TATA promoters. Finally, we did not consider 4 genes whose curvature was too low to be estimated accurately given the accuracy of fitness measurements ($|f''(p^*)p^{*2} < 0.006$). This leaves 21 genes for the analysis.

To estimate the local curvature at wild-type $\log_{10}$ expression x_{wt}, we focus on fitness measurements located within one order of magnitude of x_{wt}: $x_{wt} - 1 < x < x_{wt} + 1$.

To these measurements, we then fit the parameters a, c, d of the polynomial:

$$f(x) = a + \frac{c}{2!}(x - x_{wt})^2 + \frac{d}{3!}(x - x_{wt})^3. \tag{6}$$

There is no first order term because fitness peaks at wild-type expression. The third order term allows for asymmetry in the fitness function. c is the curvature of $f(x)$ at x_{wt}. Using Eq. 5 for the curvature of fitness as a function of $\log_{10} p$, we find a relationship between the curvature and the c parameter of the polynomial:

$$p^{*2} f''(p^*) = \frac{c}{\log(10)^2}. \tag{7}$$

We estimate the standard error on c using Fisher's information matrix, assuming a 10% error on fitness measurements[22].

By rewriting Eq. 55 of the main text for the optimal β_p/β_m in terms of $f''(p^*)p^{*2}$, we can predict the local curvature of fitness functions from the precision - economy theory:

$$f''(p^*)p^{*2} = -2\beta_m^2 l_m \frac{c_m}{\alpha_p}. \tag{8}$$

We estimate the error on these predictions by considering that the sampling error on $\log_{10} \beta_m$ is about 0.1 (see section on data processing), and a 2-fold error on c_m (mainly due to uncertainty in the number of mRNAs per cell). This leads to a standard error of 0.36 on the $\log_{10}$ predictions.

We find that predictions and measurements of $|f''(p^*)|p^{*2}$ span the same range (10^{-3} – 10^{-1}). The correlation coefficient between predictions and measurements is positive ($r = 0.39$).

To determine the significance of the agreement between predictions and measurements, we compute the root-mean-square deviation (RMSD) between predictions and measurements. Upon shuffling the measurements 10^6 times, only in $p = 4.1\%$ of shuffles is the RMSD smaller than the RMSD computed on the non-shuffled measurements. Hence, the agreement between predictions and measurements is unlikely explained by chance. Consistent with this result, a χ^2 test concludes that predictions and measurements do not differ significantly given the measurement error ($p = 0.51$).

The region in which transcription noise is comparable to the noise floor does not correspond to the depleted region

The depleted region cannot be explained by determining the β_m and β_p for which increasing transcription provides little extra precision relative to the the noise floor.

To see why, consider Eq. 3 of the main text, which relates transcription β_m to the noise σ/p:

$$\frac{\sigma^2}{p^2} = \frac{1}{p} + \frac{\alpha_p}{\beta_m} + c_{v0}^2. \tag{9}$$

Transcription noise becomes comparable to the noise floor when $\frac{\alpha_p}{\beta_m} \simeq c_{v0}^2$. Thus, if the depleted region corresponds to combinations of β_m and β_p for which transcription provides little extra precision compared to the noise floor, the boundary of the depleted region should be

$$\beta_m < \frac{\alpha_p}{c_{v0}^2}. \tag{10}$$

Therefore, the predicted boundary is a vertical line in the Crick space. This is a bad model for the data. For example, in *S. cerevisiae*, the predicted boundary would be $\log_{10}\beta_m = 1.6$ which excludes about half of the genome (Fig. 2A).

Identifying groups of genes with significantly high or low translation / transcription ratio

Do genes with high or low translation / transcription ratio have different biological functions? To find out, one could iterate through groups of genes of similar function — as defined by Gene Ontology (GO) annotations[48] — and test statistically whether genes in these groups show significantly high or low β_p/β_m. However, this approach neglects a key property of the central dogma rates: high abundance proteins cannot

have high translation / transcription ratios due to the maximal translation rate β_p^{max}. Since translation rates β_p cannot exceed β_p^{max}, achieving high protein abundance requires recruiting transcription, which decreases β_p/β_m.

As a result, stratifying genes by equal β_p/β_m arbitrarily groups low abundance proteins with low β_p/β_m together with high abundance proteins that would have high β_p/β_m but cannot because translation rates cannot exceed β_p.

To address this issue, we stratify genes by their position relative to two boundaries: the line of lowest possible translation / transcription ratios ($\beta_p = k\beta_m$), and the line of highest possible translation rates ($\beta_p = \beta_p^{max}$). We summarize the position of genes in between these two boundaries by an angle θ (Supplementary Fig. 5C). Genes that sit on the line of lowest possible translation / translation ratios have $\theta = 0$, whereas $\theta = \pi/4$ corresponds to genes with maximal translation / transcription ratios.

θ can be computed from a gene's β_m and β_p, and from the maximal transcription and translation rates β_m^{max} and β_p^{max} by trigonometry (Supplementary Fig. 5C):

$$\tan\eta = \frac{\beta_p^{max} - \beta_p}{\beta_m^{max} - \beta_m}. \tag{11}$$

The line of lowest possible translation / transcription ratio has slope 1 ($\beta_p = k\beta_m$), $\eta + \theta = \pi/4$. Therefore,

$$\theta = \pi/4 - \tan^{-1}\frac{\beta_p^{max} - \beta_p}{\beta_m^{max} - \beta_m}. \tag{12}$$

For each group of genes from the Gene Ontology, we use the Mann-Whitney test to compute the p-value that the θs in that group are significantly larger or smaller compared to the distribution of θs in genes overall. We estimate the false discovery rates (q-values) using the fdrtool R package[49].

For all four model organisms, Supplementary Data 1 lists all GO categories with significantly high or low θ with false discovery rate $q < 0.01$, together with (uncorrected) p-values and θ normalized to $\pi/4$ so that θ ranges between 0 (for GO categories with lowest β_p/β_m) and 1 (for GO categories with highest β_p/β_m). Supplementary Table 2 shows a summary of these results. Supplementary Fig. 5D–G shows the

position of genes belonging to selected significant GO categories in the Crick space.

Differences in the genes groups with high and low β_p/β_m in HeLa compared to 3T3 cells can be explained by global differences in gene expression profiles.

Different groups of genes take unusually high or low β_p/β_m in HeLa cells compared to 3T3 cells (Supplementary Table 2). Here we examine whether these differences can be explained by global differences in the gene expression.

We obtained $\log_2$ RPKMs for HeLa and 3T3 cells the RNAseq experiments of Eichhorn et al.[7]. Because 3T3 cells are of murine origin, comparing the two cell lines requires mapping mouse gene names to human names. To so so, we used ENSEMBL's orthology database, using only one-to-one orthology relations of highest confidence (confidence = 1). After mapping genes names from mouse to human, we discarded 31 non-unique genes.

To provide more context for the gene expression analysis, we merged the HeLa and 3T3 gene expression profiles with the Cancer Cell Line Encyclopedia (CCLE) gene expression data[50] based on human gene names. We excluded haematopoietic and lymphoid cell lines which form a distinct group of liquid tumors compared to the majority of solid tumor cell lines in this dataset. We quantile normalized the gene expression data of all the cell lines together.

To visualize the position of HeLa and 3T3 cell lines relative to other cancer cell lines, we performed principal component analysis on the log gene expression matrix of 859 cell lines x 6300 genes. The position of the different cell lines (gray dots) in the gene expression space spanned by first 3 PCs is shown on Supplementary Fig. 5H.

Finally, we determined gene sets that were differently regulated in HeLa compared to 3T3 by gene set enrichment analysis[51] (Supplementary Fig. 5I).

Simulating different target size of mutations affecting transcription and translation cannot explain the depleted region

An alternative hypothesis to explain the depleted region of Crick could be that mutations affecting transcription and translation to tune protein protein abundance occur with different frequency. For example, mutational target sizes could be different for transcription and translation.

To test whether a difference in target size between mutations that affect transcription or translation could explain the depleted region, we perform an evolutionary simulation.

For each gene whose evolution we simulate, we initialize the transcription rate β_m and translation rate β_p by sampling from a uniform distribution bounded by the range of transcription and translation rates found in *E. coli* (see inset titled 'Gen. 1' in Supplementary Fig. 5K).

We first pick an optimal protein abundance p^* by sampling the protein abundance of *E. coli* genes found by Li et al.[4]. The fitness of each gene is defined as

$$e^{-\frac{1}{2}\left(\frac{\log_{10}(\beta_m\beta_p/\alpha_m\alpha_p)-\log_{10}p^*}{\sigma}\right)^2}$$

with $\sigma = 0.25$. Thus, fitness is maximal when β_m and β_p are such that protein abundance is p^*, and decreases with a typical abundance scale of a quarter order of magnitude.

The evolutionary simulation alternates rounds of mutations and of selection. In each mutation round, we first determine whether the mutation would affect transcription or translation by random sampling. The odds r of the mutation affecting translation vs transcription is a control parameter. r represents the ratio between the target size of mutations that affect translation over the target size of mutations that affect transcription. We set r to $0.001, 0.01, 0.1, 1, 10, 100, 1000$. Supplementary Fig. 5K shows simulations results for $r = 10$ and $r = 100$ in favor of mutations that affect translation.

Having determined whether the mutation would affect transcription or translation, the corresponding rate $\log_{10}\beta_m$ or $\log_{10}\beta_p$ is then perturbed by adding noise drawn from a Gaussian distribution of mean 0 and variance 1. Thus, the typical mutation perturbs transcription or translation by an order of magnitude. Mutations that push transcription or translation outside the range of values found in *E. coli* are rejected. Mutations that do not improve fitness are also rejected.

The mutation and selection step define a generation in the simulation. Simulations are run for 10^4 generations. At each generation, we simulate the parallel evolution of 3744 genes, the number of genes for which transcription and translation was measured by Li et al.[4]. We monitor the average fitness at each generation to verify that evolution converged after 10^4 generations (see insets in Supplementary Fig. 5K).

Simulations show that different mutation target size ratios do not create a depleted region (Fig. 5K).

The distribution in simulated Crick space is along a diagonal that is nearly orthogonal to the distribution of natural gene (Fig. 2D). We conclude that target size alone is not a viable explanation for the depleted region.

Supplementary References

1. Weinberg, D. E. et al. Improved Ribosome-Footprint and mRNA Measurements Provide Insights into Dynamics and Regulation of Yeast Translation. *Cell Reports* **14**, 1787–1799 (2016). http://dx.doi.org/10.1016/j.celrep.2016.01.043.
2. Eser, P. et al. Periodic mRNA synthesis and degradation co-operate during cell cycle gene expression. *Molecular systems biology* **10**, 717 (2014).
3. Belle, A. et al. Quantification of protein half-lives in the budding yeast proteome. *Proceedings of the National Academy of Sciences of the United States of America* **103**, 13004–9 (2006). http://dx.doi.org/10.1073/pnas.0605420103.
4. Li, G.-W., Burkhardt, D., Gross, C. & Weissman, J. S. Quantifying Absolute Protein Synthesis Rates Reveals Principles Underlying Allocation of Cellular Resources. *Cell* **157**, 624–635 (2014). http://dx.doi.org/10.1016/j.cell.2014.02.033.
5. Chen, H., Shiroguchi, K., Ge, H. & Xie, X. S. Genome-wide study of mRNA degradation and transcript elongation in Escherichia coli. *Molecular Systems Biology* **11**, 781–781 (2015). http://dx.doi.org/10.15252/msb.20145794.
6. Schwanhüusser, B. et al. Global quantification of mammalian gene expression control. *Nature* **473**, 337–342 (2011). http://dx.doi.org/10.1038/nature10098.
7. Eichhorn, S. W. et al. mRNA Destabilization Is the Dominant Effect of Mammalian MicroRNAs by the Time Substantial Repression Ensues. *Molecular cell* **56**, 104–15 (2014). http://dx.doi.org/10.1016/j.molcel.2014.08.028.
8. Cambridge, S. B. et al. Systems-wide proteomic analysis in mammalian cells reveals conserved, functional protein turnover. *Journal of proteome research* **10**, 5275–84 (2011). http://dx.doi.org/10.1021/pr101183k.
9. Baba, T. et al. Construction of Escherichia coli K-12 in-frame, single-gene knockout mutants: the Keio collection. *Molecular systems biology* **2**, 2006.0008 (2006). http://dx.doi.org/10.1038/msb4100050.
10. Nagaraj, N. et al. Deep proteome and transcriptome mapping of a human cancer cell line. *Molecular*

systems biology **7**, 548 (2011). http://dx.doi.org/10.1038/msb.2011.81.

11. Newman, J. R. S. et al. Single-cell proteomic analysis of S. cerevisiae reveals the architecture of biological noise. *Nature* **441**, 840–6 (2006). http://dx.doi.org/10.1038/nature04785.

12. Friedman, N., Cai, L. & Xie, X. S. Linking Stochastic Dynamics to Population Distribution: An Analytical Framework of Gene Expression. *Physical Review Letters* **97**, 168302 (2006). http://dx.doi.org/10.1103/PhysRevLett.97.168302.

13. Friedman, N., Cai, L. & Xie, X. S. Stochasticity in Gene Expression as Observed by Single-molecule Experiments in Live Cells. *Israel Journal of Chemistry* **49**, 333–342 (2009). http://dx.doi.org/10.1560/IJC.49.3\OT1\textendash4.333.

14. Li, J. J., Bickel, P. J. & Biggin, M. D. System wide analyses have underestimated protein abundances and the importance of transcription in mammals. *PeerJ* **2**, e270 (2014). http://dx.doi.org/10.7717/peerj.270.

15. Kosuri, S. et al. Composability of regulatory sequences controlling transcription and translation in Escherichia coli. *Proceedings of the National Academy of Sciences of the United States of America* **110**, 14024–9 (2013). http://dx.doi.org/10.1073/pnas.1301301110.

16. Sharon, E. et al. Probing the effect of promoters on noise in gene expression using thousands of designed sequences. *Genome Research* **24**, 1698–1706 (2014). http://dx.doi.org/10.1101/gr.168773.113.

17. Friedman, R. C., Farh, K. K.-H., Burge, C. B. & Bartel, D. P. Most mammalian mRNAs are conserved targets of microRNAs. *Genome research* **19**, 92–105 (2009). http://dx.doi.org/10.1101/gr.082701.108.

18. Ingolia, N. T., Ghaemmaghami, S., Newman, J. R. S. & Weissman, J. S. Genome-wide analysis in vivo of translation with nucleotide resolution using ribosome profiling. *Science* **324**, 218–23 (2009). http://dx.doi.org/10.1126/science.1168978.

19. Taniguchi, Y. et al. Quantifying E. coli proteome and transcriptome with single-molecule sensitivity in single cells. *Science* **329**, 533–538 (2010). http://dx.doi.org/10.1126/science.1188308.

20. So, L.-H. H. et al. General properties of transcriptional time series in Escherichia coli. *Nature genetics*

43, 554–60 (2011). http://dx.doi.org/10.1038/ng.821.

21. Gout, J.-F. et al. The landscape of transcription errors in eukaryotic cells. *Science advances* **3**, e1701484 (2017). http://dx.doi.org/10.1126/sciadv.1701484.

22. Keren, L. et al. Massively Parallel Interrogation of the Effects of Gene Expression Levels on Fitness. *Cell* **166**, 636–643 (2016). http://dx.doi.org/10.1016/J.CELL.2016.07.024.

23. Silander, O. K. et al. A genome-wide analysis of promoter-mediated phenotypic noise in Escherichia coli. *PLoS genetics* **8**, e1002443 (2012). http://dx.doi.org/10.1371/journal.pgen.1002443.

24. Steinmetz, L. M. et al. Systematic screen for human disease genes in yeast. *Nature genetics* **31**, 400–4 (2002). http://dx.doi.org/10.1038/ng929.

25. Molina-Quiroz, R. C. et al. Cyclic AMP Regulates Bacterial Persistence through Repression of the Oxidative Stress Response and SOS-Dependent DNA Repair in Uropathogenic Escherichia coli. *mBio* **9**, e02144–17 (2018). http://dx.doi.org/10.1128/mBio.02144-17.

26. Vega, N. M., Allison, K. R., Khalil, A. S. & Collins, J. J. Signaling-mediated bacterial persister formation. *Nature Chemical Biology* **8**, 431–433 (2012). http://dx.doi.org/10.1038/nchembio.915.

27. Kussell, E. & Leibler, S. Phenotypic diversity, population growth, and information in fluctuating environments. *Science (New York, N.Y.)* **309**, 2075–8 (2005). http://dx.doi.org/10.1126/science.1114383.

28. Levy, S. F., Ziv, N. & Siegal, M. L. Bet Hedging in Yeast by Heterogeneous, Age-Correlated Expression of a Stress Protectant. *PLoS Biology* **10**, e1001325 (2012). http://dx.doi.org/10.1371/journal.pbio.1001325.

29. Albers, E. et al. Effect of nutrient starvation on the cellular composition and metabolic capacity of Saccharomyces cerevisiae. *Applied and environmental microbiology* **73**, 4839–48 (2007). http://dx.doi.org/10.1128/AEM.00425-07.

30. Gray, J. V. et al. ”Sleeping beauty”: quiescence in Saccharomyces cerevisiae. *Microbiology and molecular biology reviews* **68**, 187–206 (2004). http://dx.doi.org/10.1128/MMBR.68.2.187-206.2004.

31. Basan, M. et al. Overflow metabolism in Escherichia coli results from efficient proteome allocation. *Nature* **528**, 99–104 (2015). http://dx.doi.org/10.1038/nature15765.

32. Parker, G. A. & Smith, J. M. Optimality theory in evolutionary biology. *Nature* **348**, 27–33 (1990). http://dx.doi.org/10.1038/348027a0.

33. Scott, M. et al. Interdependence of Cell Growth and Gene Expression: Origins and Consequences. *Science* **330**, 1099–1102 (2010). http://dx.doi.org/10.1126/science.1192588.

34. Towbin, B. D. et al. Optimality and sub-optimality in a bacterial growth law. *Nature Communications* **8**, 14123 (2017). http://dx.doi.org/10.1038/ncomms14123.

35. Savir, Y. & Tlusty, T. The ribosome as an optimal decoder: a lesson in molecular recognition. *Cell* **153**, 471–479 (2013). http://dx.doi.org/10.1016/j.cell.2013.03.032.

36. Proshkin, S., Rahmouni, A. R., Mironov, A. & Nudler, E. Cooperation Between Translating Ribosomes and RNA Polymerase in Transcription Elongation. *Science* **328** (2010).

37. Gupta, I. et al. Translational Capacity of a Cell Is Determined during Transcription Elongation via the Ccr4-Not Complex. *Cell Reports* **15**, 1782–1794 (2016). http://dx.doi.org/10.1016/j.celrep.2016.04.055.

38. Presnyak, V. et al. Codon optimality is a major determinant of mRNA stability. *Cell* **160**, 1111–1124 (2015). http://dx.doi.org/10.1016/j.cell.2015.02.029.

39. Oxman, E., Alon, U. & Dekel, E. Defined order of evolutionary adaptations: experimental evidence. *Evolution* **62**, 1547–54 (2008). http://dx.doi.org/10.1111/j.1558-5646.2008.00397.x.

40. Wiser, M. J., Ribeck, N. & Lenski, R. E. Long-Term Dynamics of Adaptation in Asexual Populations. *Science* **342**, 1364–1367 (2013). http://dx.doi.org/10.1126/science.1243357.

41. Scherer, W. F., Syverton, J. & Gey, G. Studies on the Propagation in Vitro of Poliomyelitis Viruses: Iv. Viral Multiplication in a Stable Strain of Human Malignant Epithelial Cells (Strain Hela) Derived From an Epidermoid Carcinoma of the Cervix. *Journal of Experimental Medicine* **97**, 695–710 (1953). http://dx.doi.org/10.1084/jem.97.5.695.

42. Todaro, G. J. & Green, H. Quantitative studies of the growth of mouse embryo cells in culture and their development into established lines. *The Journal of Cell Biology* **17**, 299–313 (1963).

43. Wu, S. et al. Independent regulation of gene expression level and noise by histone modifications. *PLOS Computational Biology* **13**, e1005585 (2017). http://dx.doi.org/10.1371/journal.pcbi.1005585.

44. Weinberger, L. et al. Expression Noise and Acetylation Profiles Distinguish HDAC Functions. *Molecular Cell* **47**, 193–202 (2012). http://dx.doi.org/10.1016/J.MOLCEL.2012.05.008.

45. Paulsson, J. Models of stochastic gene expression. *Physics of Life Reviews* **2**, 157–175 (2005). http://dx.doi.org/10.1016/j.plrev.2005.03.003.

46. Dar, R. D. et al. Transcriptional burst frequency and burst size are equally modulated across the human genome. *Proceedings of the National Academy of Sciences of the United States of America* **109**, 17454–9 (2012). http://dx.doi.org/10.1073/pnas.1213530109.

47. Zenklusen, D., Larson, D. R. & Singer, R. H. Single-RNA counting reveals alternative modes of gene expression in yeast. *Nature structural & molecular biology* **15**, 1263–1271 (2008). http://dx.doi.org/10.1038/nsmb.1514.

48. Ashburner, M. et al. Gene ontology: tool for the unification of biology. The Gene Ontology Consortium. *Nature genetics* **25**, 25–9 (2000).

49. Strimmer, K. fdrtool: a versatile R package for estimating local and tail area-based false discovery rates. *Bioinformatics* **24**, 1461–1462 (2008). http://dx.doi.org/10.1093/bioinformatics/btn209.

50. Barretina, J. et al. The Cancer Cell Line Encyclopedia enables predictive modelling of anticancer drug sensitivity. *Nature* **483**, 603–307 (2012). http://dx.doi.org/10.1038/nature11003.

51. Subramanian, A. et al. Gene set enrichment analysis: a knowledge-based approach for interpreting genome-wide expression profiles. *Proceedings of the National Academy of Sciences of the United States of America* **102**, 15545–50 (2005). http://dx.doi.org/10.1073/pnas.0506580102.
