# Supplementary material for: Central dogma rates and the trade-off between precision and economy in gene expression
Source: Nat Commun. 2019 Jan 8;10:68. doi: 10.1038/s41467-018-07391-8 (PMC6325141; doi:10.1038/s41467-018-07391-8)
Supplement: Supplementary file 3 — Description of Additional Supplementary Files [file 41467_2018_7391_MOESM3_ESM.pdf]

Description of Additional Supplementary Files

File Name: Supplementary Data 1
Description: Enriched GO terms in genes with high and low bp/bm ratios
